# Co-Evaporated CuO-Doped In_2_O_3_ 1D-Nanostructure for Reversible CH_4_ Detection at Low Temperatures: Structural Phase Change and Properties

**DOI:** 10.3390/ma12244073

**Published:** 2019-12-06

**Authors:** N.M. Shaalan, D. Hamad, Osama Saber

**Affiliations:** 1 Department of Physics, College of Science, King Faisal University, Al-Hassa 31982, Saudi Arabia; 2 Physics Department, Faculty of Science, Assiut University, Assiut 71516, Egypt; noniatypes@yahoo.com; 3 Egyptian Petroleum Research Institute, Nasr 11727, Egypt

**Keywords:** copper-doped tin oxide, thermal evaporation, 1D-nanostructures, methane gas sensor

## Abstract

In order to improve the sensitivity and to reduce the working temperature of the CH_4_ gas sensor, a novel 1D nanostructure of CuO-doped In_2_O_3_ was synthesized by the co-evaporation of Cu and In granules. The samples were prepared with changing the weight ratio between Cu and In. Morphology, structure, and gas sensing properties of the prepared films were characterized. The planned operating temperatures for the fabricated sensors are 50–200 °C, where the ability to detect CH_4_ at low temperatures is rarely reported. For low Cu content, the fabricated sensors based on CuO-doped In_2_O_3_ showed very good sensing performance at low operating temperatures. The detection of CH_4_ at these low temperatures exhibits the potential of the present sensors compared to the reported in the literature. The fabricated sensors showed also good reversibility toward the CH_4_ gas. However, the sensor fabricated of CuO-mixed In_2_O_3_ with a ratio of 1:1 did not show any response toward CH_4_. In other words, the mixed-phase of p- and n-type of CuO and In_2_O_3_ materials with a ratio of 1:1 is not recommended for fabricating sensors for reducing gas, such as CH_4_. The gas sensing mechanism was described in terms of the incorporation of Cu in the In_2_O_3_ matrix and the formation of CuO and In_2_O_3_ phases.

## 1. Introduction

It is known that CH_4_ is detected by a few materials at high operating temperatures. However, some literature reported developed materials that give a satisfactory performance to CH_4_ at relatively high temperatures [1,2,3,4,5,6,7,8,9]. Among these materials, SnO_2_ NR–NP-Gr hybrids and PdPt-SnO_2_ -rGO which showed a promising property for methane at as low as 150 °C [2,10]. Also, our previous In_2_O_3_ nanostructure film showed a promising performance toward methane at 100 °C [11]. As technology improved, an E-nose for CH_4_ at RT was developed by commercially available SnO_2_ [12]. However, the operating temperature of the promising In_2_O_3_ sensor needs to be reduced to lower temperatures.

Recently, researchers have paid great attention to develop new nanocomposites of binary or ternary metal oxides with a new functionality [13,14,15,16,17]. Heterostructure materials offer more opportunities by the effective combination of two or more chemically dissimilar components in a single nanostructure with multifunctional properties. They are surely led to an innovative application of nanomaterials in various fields. Thus, the creation of heterostructure materials is an effective way to achieve low-temperature gas sensor [18,19,20,21]. N-type In_2_O_3_ is a wide bandgap semiconductor with stable physical and chemical properties. Its thin film is important for many applications and it is transparent to the visible light. Currently, In_2_O_3_ films are used for solar cells [22], optoelectronic devices [23], and transparent conducting oxide [24]. Undoped In_2_O_3_ has been used for gas sensors [25,26,27]. It was used by many structures prepared by various methods and precursors [28,29,30]. Undoped In_2_O_3_ showed promising performance for detecting gases, such as H_2_ [31], NO_2_ [32], and CH_4_ [11,33]. Also, p-type CuO owns specific structural properties with a monoclinic stable phase [34]. Researchers have been attracted by its sensing properties toward reducing gas [35]. Thus, the enhancement of In_2_O_3_ sensitivity to CH_4_ is expected if it is mixed or doped with proper materials.

CuO and In_2_O_3_ nanoscale materials have been investigated for gas sensing applications [11,35], showing promising properties. However, no reports have focused on the CuO-doped In_2_O_3_ materials to CH_4_ gas detection. In this paper, we intend to study the impact of co-evaporation of CuIn on the structural and CH_4_ sensing of Cu-doped In_2_O_3_ with low and high Cu content to develop a low-temperature gas sensor for CH_4_. CuO-mixed In_2_O_3_ nanocomposite films are synthesized under a low vacuum in the presence of partial oxygen. The prepared films are characterized for structure and morphology using X-ray diffractometer, Raman spectroscopy, EDX techniques, and scanning electron microscope. The evaluation of the methane sensing properties of the synthesized gas sensors by the present composites is well investigated at low operating temperatures.

## 2. Experimental Details

### 2.1. Preparation Method

The preparation of films was carried out in a vacuum stainless-steel chamber. The evaporation takes place in the evaporation closed system consisted of alumina crucible surrounded by the heating element and covered by a glass substrate. The deposition area is limited to an exposed area of 6.28 cm^2^, keeping the substrate is heated. The temperature of the set is controlled at 900 °C by the supplied current to the heater.

### 2.2. Co-Evaporation and In Situ Oxidation of Nanocomposite

To prepare CuO-mixed In_2_O_3_, Cu, and In pieces of total 3.0 mg with a purity of 99.999% are kept in the evaporation source, which covered by the glass substrate connected with a thermocouple. The chamber was kept under a vacuum of 7 × 10^−2^ mbar, keeping the air is flowing into the chamber, which is connected to a rotary pump. Then the specific current was applied on the heating element to heat up the evaporation set up to 900 °C within 5.0 min, which was kept for 30 min, where the temperature of the substrate is maintained at 400 °C. Afterward, the evaporation set is naturally cooled down to room temperature, taking about 90 min to cool. After deposition, it is found that the substrate is coated with a film strongly adhered to the substrate. The thickness of the deposited film was about 1 µm. The color of the deposited film is depending on the ratio Cu:In, where it was light yellowish for In_2_O_3_ and then came to be black with increasing Cu content. Three samples are prepared based on the weight ratio in mg of Cu:In granules as 0.5:2.5, 1.0:2.0, and 2.5:0.5, which are assigned here as S1, S2, and S3, respectively.

### 2.3. Characterization Tools

CuKα X-ray technique (Philips Type PW 1710) with 1.546 Å was used to investigate the crystal structure of synthesized nanocomposites in diffraction angle (2Ɵ = 4–70°). the morphology was observed by using SEM (JEOL JSM-5400LV) (Tokyo, Japan) attached with EDX for elemental analysis of the formed composites. The nanostructured films were also characterized by Raman spectroscopy. Raman shift spectra were recorded from the film samples with FRA 106 (Nd: YAG-Laser. 442 nm) spectrometer.

### 2.4. Sensor Preparation and Characterization

**Figure 1:**
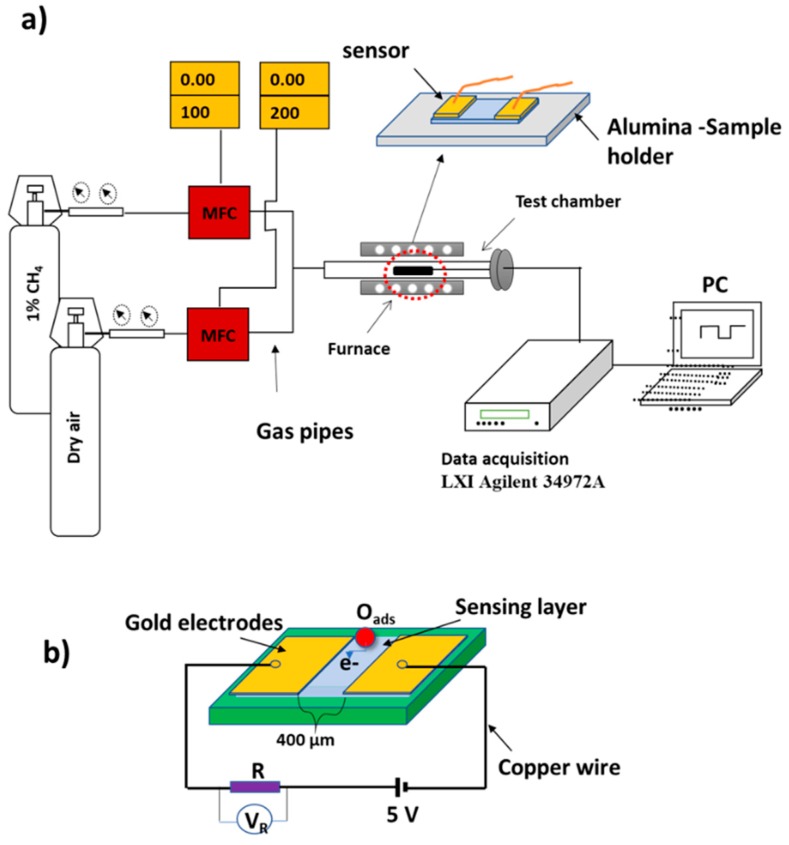
(**a**) Schematic illustration of the gas sensor measurement equipment with the sample stage. (**b**) Schematic diagram of the device and electrical connections.

For fabricating the gas sensor, sputtered two electrodes of gold with a gap of 400 µm were deposited on the surface of the sensing layer at room temperature (RT). Because the sensors are investigated at operating temperatures higher than RT (up to 200 °C), and as a providence process, they were treated at 350 °C for 30 min in the air in order to avoid any change in contact interface during the sensing measurements. Figure 1 shows the schematic illustration of the gas sensing equipment and the sample stage. The sensing measurements were performed in a quartz chamber, which consists of a controlled electric furnace and electrical terminals connected to a computerized data acquisition (Multi-channel LXI Agilent 34972 A). The low operating temperature applied here lies between 50 and 200 °C. Three sensors are applied to the same conditions where a dry synthetic air (21% O_2_ + 79% N_2_) mixed with 1.0% CH_4_ is flowing with 200 SCCM into the testing chamber by using SEC-N112 MGM-Horiba. Figure 1b shows the schematic diagram of the device with the electrical connections. The resistance and output voltage of the sensors is calculated with applying of 5.0-V potential difference to the electric circuit.

## 3. Results and Discussions

### 3.1. Structural Characterizations

**Figure 2:**
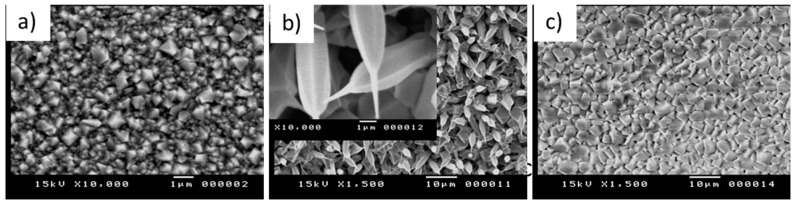
SEM images of the sample prepared at various Cu content of Cu:In; (**a**) S1, (**b**) S2, and (**c**) S3.

The morphology of the films obtained of the co-evaporated Cu and In is characterized by SEM images, as shown in Figure 2. The slow evaporation or sublimation of the mixed-phase allows the nanostructures to grow up. The grain-like structure is observed for S1 and S3, whereas, the 1D nanostructure is observed for S2. The surface thermal energy of the heated substrate allows the landing atoms to aggregate and build up nuclei for growing nanostructure. In earlier work, a pure phase of In_2_O_3_ prepared under the same condition was sphere-like grains [11]. Thus, the incorporation of Cu atoms in the present evaporation may affect the dimension growth. With low Cu content, the seeds of the one-dimensional structure started as shown in the S1 sample. With moderated Cu content, 1D nanostructures are growing, as shown in the S2 sample (Figure 2b). The 1D nanostructure is inclined or vertical to the substrate surface and ended with needle-like at the tips. It seems that the excessive Cu content in the composite suppressed the growth of the 1D-nanostructure, as shown in the S3 sample (Figure 2c).

**Figure 3:**
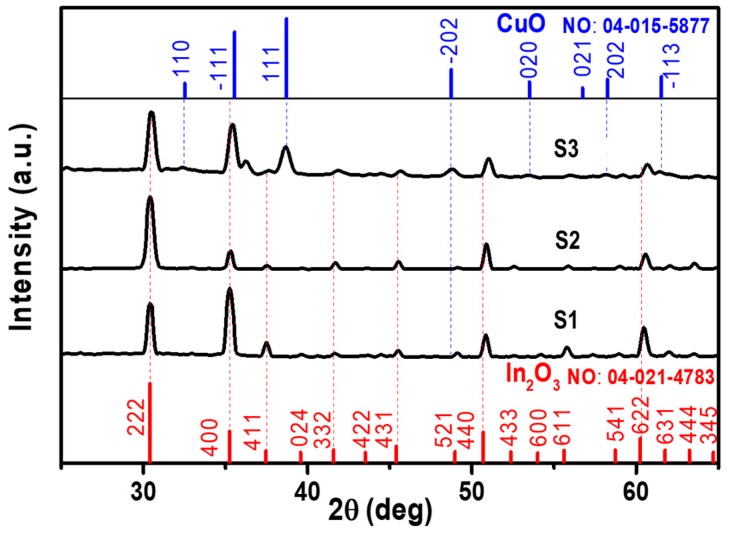
XRD charts for the sample prepared at various Cu contents. (**a**) S1, (**b**) S2, and (**c**) S3.

Figure 3 illustrates the XRD charts of the prepared nanostructures for S1, S2, and S3 with adopted CuO (no. 04-015-5877) and In_2_O_3_ (no. 04-021-4783). The analysis of XRD showed that the films are polycrystalline with a cubic bixbyite-type structure In_2_O_3_ and monoclinic structure of CuO. It is observed that the preferred orientation planes are (222) for In_2_O_3_ and (−111) and (111) for CuO. From XRD charts several observations can be concluded. The first observation is the shift in the peaks (222), (400), (440), (622), and (444) to high diffraction angles, indicating the incorporation of Cu atoms into the In_2_O_3_ matrix. By comparing the ionic radii of In^3+^ (0.81 Å) and Cu^2+^ (0.73 Å), the substitution is expected mechanism for incorporating Cu^2+^ ions into In_2_O_3_ lattice at low concentrations. The substitution causes a shrinkage in the lattice dimensions, which is observed from the shift of peak positions to large diffraction angles. The second observation is the appearance of new peaks of (110), (111), and (−202) which may be close to the CuO phase. They have a shift also to the large diffraction angles. Nonetheless, it is expected for CuO peak positions to be shifted to lower diffraction angles because d-spacing should expand due to In^3+^ radii. A final observation is the dominance of the orientation plane of (222) for the sample S2 overall other orientations, confirming the formation of vertical-like structure observed for S2. From these observations, we can claim that a new phase nanocomposite of CuO-mixed In_2_O_3_ is formed. Celref UNIT CELL software was used to calculate the lattice parameters using the raw data obtained from XRD. From the XRD chart analysis, the lattice parameters and d-spacing of In_2_O_3_ shrinkage respective to the standard values recorded in card no. 04-021-4783 are listed in Table 1.

**Figure 4:**
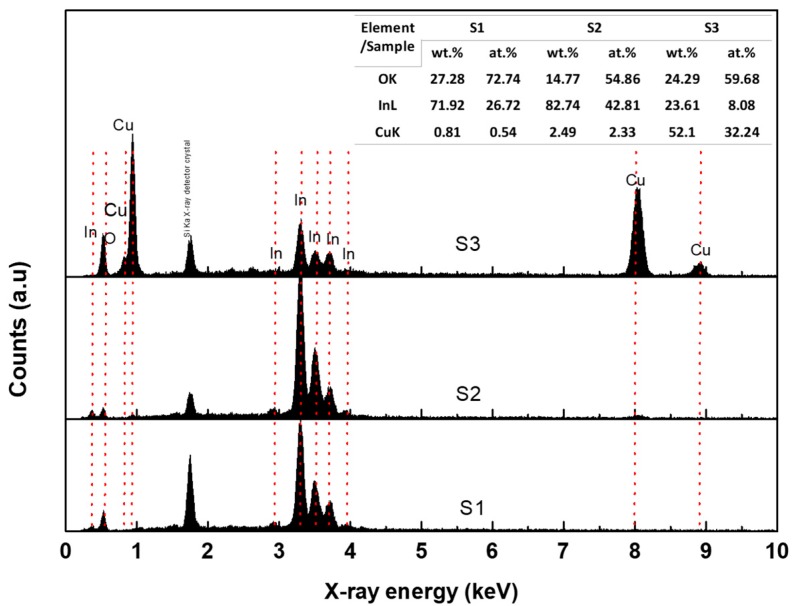
EDX spectra for the sample prepared at various Cu contents. (**a**) S1, (**b**) S2, and (**c**) S3. The table attached in the figure shows the weight and atomic percentage of the OK, CuK, and InL emission.

For supporting the data observed in XRD, EDX spectra of the obtained nanostructures are shown in Figure 4. EDX spectra exhibited the existence of CuK line of low and high Cu content. The emission of CuK spectra is very strong at high Cu content, as shown for S3 in Figure 4. The InL spectra are dominant at low Cu content. The elemental analysis of the nanostructures of the actual weight and atomic percentage for Cu and In is listed in the table attached in Figure 4. EDX analysis shows that Cu’s weight percent in the samples increases from 0.81%, to 2.49%, up to 52.1% with increasing Cu weight for S1, S2, S3, respectively. In other words, the atomic percentage of oxygen is dependent on the nanostructure or composite obtained, where it is recorded as 72.74, 54.86, and 59.68% for S1, S2, S3, respectively. This change in oxygen percentage gives an indicator of the composite formation as follows. (1) At low Cu content, the oxygen atoms are mostly linked to the In ions in In_2_O_3_ structure, where Cu atoms involved in the In_2_O_3_ lattice are less. (2) with increasing Cu ions in In_2_O_3_ lattice with substitution mechanism, the bridging oxygen atoms are missing, and the atomic oxygen ratio decreases. Moreover, the building up for 1D nanostructure may increase the oxygen vacancies on its surface. (3) with exceeding Cu content, more substitution of Cu is expected to grow up, and oxygen ions are linked with both Cu^2+^ and In^3+^, keeping the oxygen percentage less due to increasing of Cu^2+^ content (CuO).

**Figure 5:**
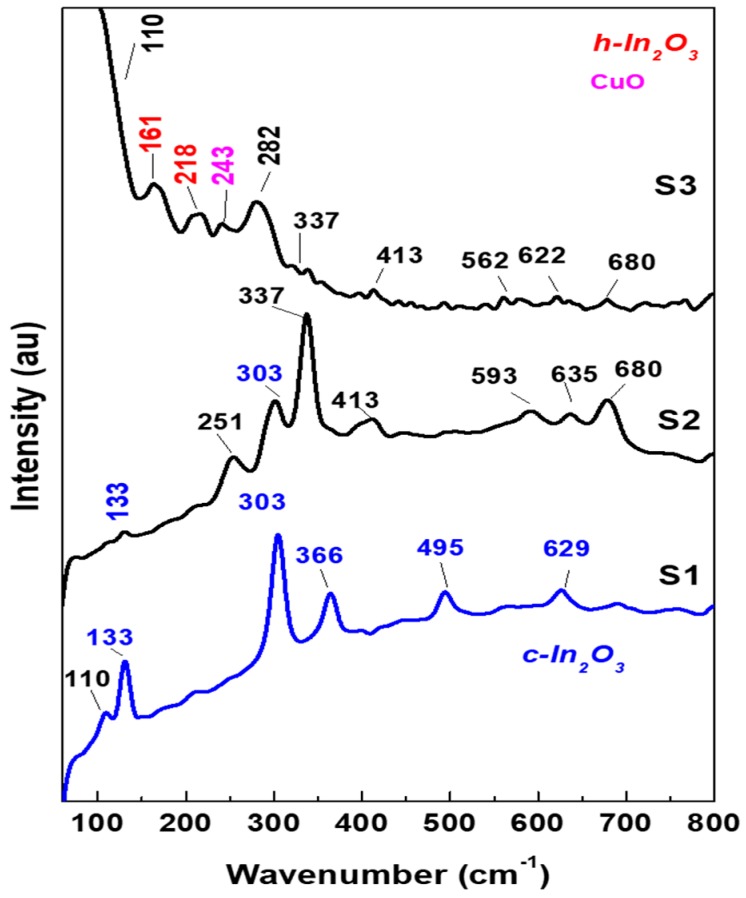
Summation of Raman scattering spectra for the sample prepared at various Cu contents. (**a**) S1, (**b**) S2, and (**c**) S3.

The measured Raman spectra of S1, S2, and S3 are shown in Figure 5. The shown spectrum for each sample is the summation of 64 spectra taken from different points on the surface in order to get a full image about the nature and the uniformity of the surface. The comparison of the measured frequencies is also listed in Table 2. For the S1 sample, where the Cu content is very less about 0.8%, the vibrations of Cu or CuO are not detected, and the only vibrations detected for cubic-In_2_O_3_. Five vibrations are observed in the range of 100–800 cm^−1^ for In_2_O_3_. The vibrations are ascribed to the vibration of [In]^3+^ cations. These vibrations are affected by the increase in the Cu content of the material. For the S2 sample, where Cu is expected to be partially substituted with In ions and form a new composite, new molecular vibrations are detected at 251, 337, 411, and 680 cm^−1^. The only vibration detected in S2 for c-In_2_O_3_ is 303 cm^−1^. With the excessive Cu up to 50% in the sample S3, new vibrations are observed at 161, 218, 243, and 281 cm^−1^. The vibrations of 161 and 218 cm^−1^ are close to the *h*-In_2_O_3_ [36], and the vibration of 243 cm^−1^ is close to CuO [37]. However, the vibration at 281 cm^−1^ may be ascribed to the new phase of CuInO_4_. The significant shift in vibrations due to the degeneracy in the crystal symmetry or the appearance of new vibrations come with an agreement with XRD data where most of the diffraction peaks of S2 and S3 are not in the original position for In_2_O_3_ and CuO, as well. The result is an evidence for the formation of a new phase mixed of Cu-In oxide. However, it is expected that a separated phase of CuO and In_2_O_3_ are formed at 50% Cu.

### 3.2. CH_4_ Detection at Low Temperatures

**Figure 6:**
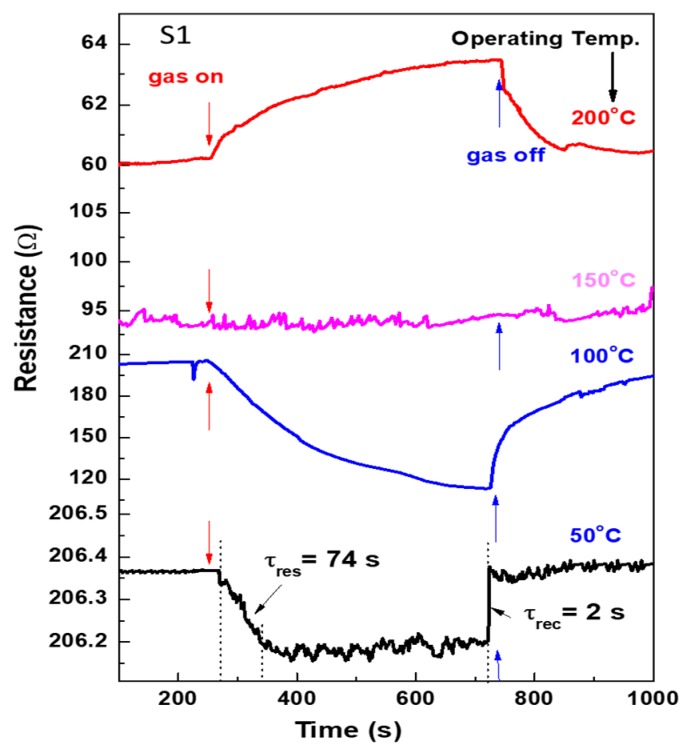
Change in resistance at operating temperatures of 50–200 °C for the sensor fabricated by the S1 sensing layer.

**Figure 7:**
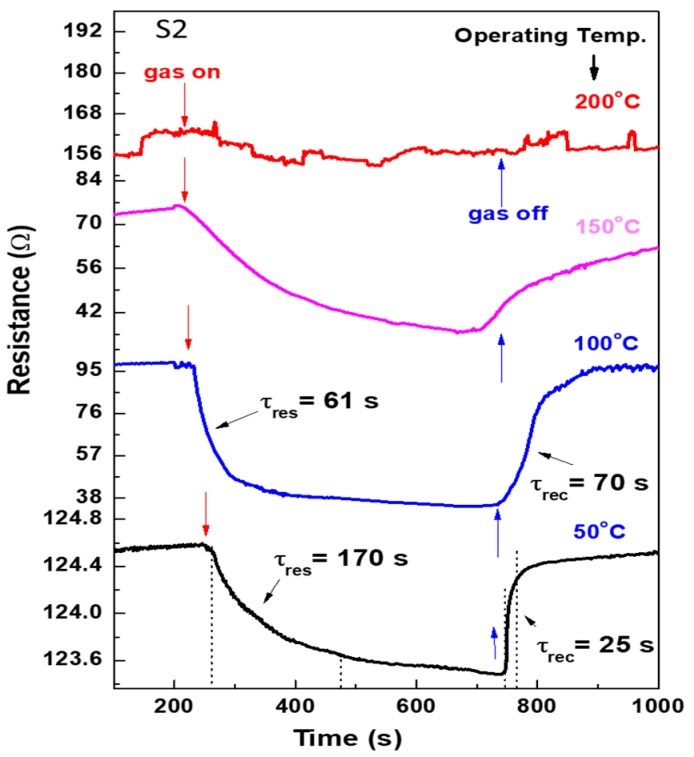
Change in resistance at operating temperatures of 50–200 °C for the sensor fabricated by the S2 layer.

**Figure 8:**
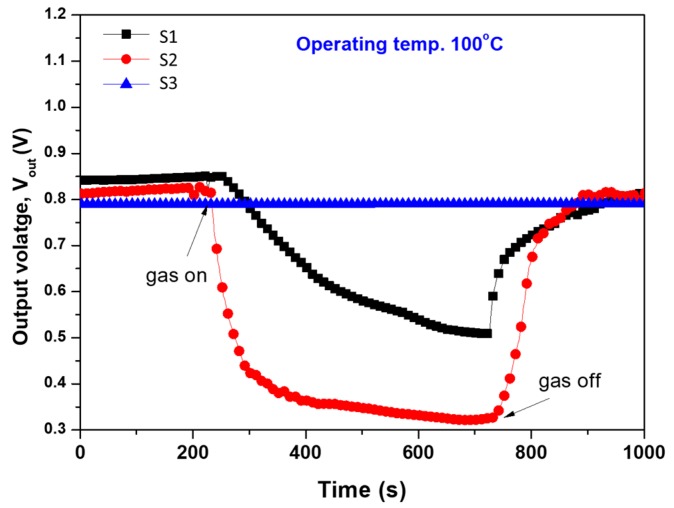
Output voltage at operating temperatures 100 °C for the sensors fabricated with S1, S2, and S3 sensing layers. Note: the sensing layer of S3 is not sensitive to methane at the operating temperature applied here (50, 150, 200 °C are not shown here).

The advantage of the mixed-phase of CuO and In_2_O_3_ is the low operating temperature sensor for methane gas. Many attempts for researchers were focused to fabricate a low-temperature methane sensor for safety control and reducing power consumption, but most of the proposed sensors are working at relatively high temperatures [1,2,3,4,5,6,7,8,9]. In the present study, we have successfully developed a sensor working at relatively low temperatures. Figure 6, Figure 7 and Figure 8 show the sensor signals toward the methane gas at low operating temperatures (50–200 °C). We should point out that the fabricated sensor did not show a response to CH_4_ at room temperature (signal not shown). Figure 6 shows the sensor signals at 50–200 °C for the S1 layer. At 50 °C, the sensor responds quickly once the methane is introduced into the measurement chamber, and it shows an n-type behavior of the semiconductor. Although the sensor response at this temperature is slightly low, the sensor recorded a response time constant about 75.0 s and recovery time constant about 2.0 s. The response/recovery time constant is the time taken to reduce or increase the sensor resistance to 90% of its initial value. The response of the sensor increased at an operating temperature of 100 °C, and then the response and recovery time constants also increased. Interesting is the behavior of the sensor at 150 °C, where the sensor does not respond to the gas at all. Then the sensor showed a behavior of p-type semiconductor at 200 °C. The resistance of the sensor increased upon exposure to the methane gas, it is due to that the sensing layer consists of n- and p-type materials.

Figure 7 shows the change in resistance for the S2 sensor at various operating temperatures, showing n-type behavior. The S2 sensor is good sensitive to methane even at a low temperature of 50 °C, the sensor response may be improved, but the response and recovery time constants slightly increased up to 170 and 25 s, respectively. On the contrary, with the S1 sensor, S2 sensor response time became shorter down to 61.0 s and the sensor response improved at 100 °C, as well. By increasing the temperature up to 150 °C, the sensor takes a long time to recover to its initial resistance. Moreover, it is insensitive to methane at 200 °C. Compared to the response time, the recovery time is small for the sensor at low temperatures. It is expected that, at such low operating temperatures, the surface is just active for gas—thus, upon the exposure to 1% gas—the reaction of gas with surface species to saturate the surface of the sensing layer is slow. However, upon the exposure to the air (switching off the gas), the desorption is fast. We may ascribe this to the weak electric force between the gas and surface species at low temperatures. This can be confirmed by the small change in the sensor resistance at 50 °C within a few ohms. Thus, the response time is longer than the recovery time.

Figure 8 shows the output voltage of sensors fabricated with S1, S2, and S3 films at 100 °C. These signals clearly distinguish between the sensing ability of the fabricated sensors, confirming that S3 is not sensitive to CH_4_.

**Figure 9:**
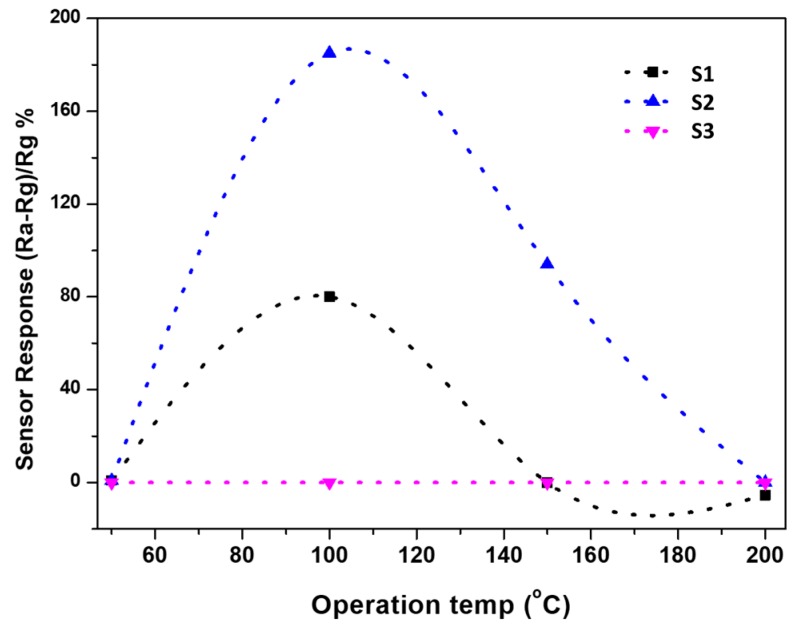
Sensor response as a function of operating temperatures for the sensors fabricated with S1, S2, and S3 sensing layers. Note: the negative values here are defined as negative sensor response for p-type behavior to be notable compared to n-type behavior.

The sensor response is calculated as

(1) $$S\%=\{\begin{array}{cc} \frac{\left(R_{a}-R_{g}\right)}{R_{g}}\times 100, & for\;n\text{-}type\;behavior\\ -\frac{\left(R_{g}-R_{a}\right)}{R_{a}}\times 100, & for\;p\text{-}type\;behavior \end{array}$$

where Ra is the resistance in air and Rg the resistance in the presence of gas. The negative sign in the definition of the sensor response differentiates between the sensor response of n- and p-type. Figure 9 shows the response as a function of the operating temperature. The sensor fabricated by S2 is showing a better sensitivity than that fabricated by S1. Although these two sensors respond to CH_4_ at 50 °C, the optimal temperature is 100 °C. The sensor fabricated by the S3 sensing layer did not show any response toward methane with increasing the operating temperature from 50 to 200 °C. It seems that the formation of p- and n-type semiconductor composite is not favored to be with 1:1 ratio. This reason can be explained through the point of view of the sensing mechanism.

### 3.3. Gas Sensing Mechanism

**Figure 10:**
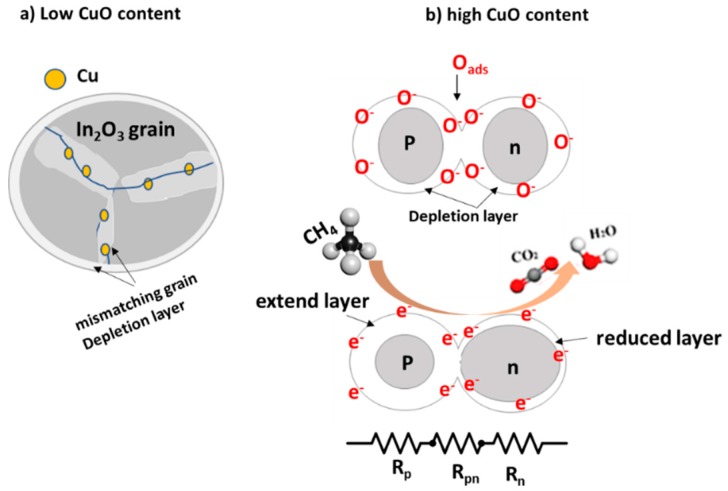
Schematic diagram of the sensing mechanism (**a**) with low Cu content, and (**b**) high Cu content of p-n type materials.

Figure 10 illustrates a schematic diagram of the gas sensing mechanism, showing two effects due to incorporating Cu into In_2_O_3_ grains and the formation of the p-CuO/n-SnO_2_ phase. As it is known, the sensing property is a surface property depending on how the gas reacts with the material. In the air, the adsorbed oxygen species create a depletion layer on the surface of oxide due to the capture of the conduction electrons. In other words, the adsorbed oxygen molecules on the surface of oxide pass through chemisorbed reaction reported by Ruhland et al. [38].

(2) $$O_{2}\left(g\right)+e^{-}\to O_{2}^{-}\gg O_{2}^{-}+e^{-}\to 2O^{-}$$

Thus, in the case of n-type materials the resistivity increases, while the resistivity decreases in the case of p-type. The incorporation of Cu^2+^ into the In_2_O_3_ matrix causes the lattice mismatching because of the difference in the ionic radii. This may cause an increase in the donor density in In_2_O_3_, allowing the adsorbed oxygen molecules to capture more electrons, which creates a depletion layer inside the grain due to the dangling bond, as proposed in Figure 10a. While the high Cu concentration formed a grain to grain interaction. In other words, p-type grain and n-type grain are formed, and it is not layered or film junction. The CuO/In_2_O_3_ heterojunction is insignificant compared to the effect of the bulk size. The expected boundary reaction between In_2_O_3_ and CuO is shown in Figure 10b. Based on this structure and the above results obtained from the gas sensing measurements, we could report the following. The current target gas is a reducing gas, which reacts with adsorbed oxygen species and produces H_2_O and CO_2_ [39,40,41].

(3) $$CH_{4}+4O_{\left(ads\right)}^{-}\to CO_{2\left(gas\right)}+2H_{2}O_{\left(gas\right)}+4e^{-}\left(\text{complete}\;\text{reaction}\right)$$

When CH_4_ gas reacts with the oxygen species, the electrons are injected back to the conduction band of the oxide. Thus, the electrons density increases in In_2_O_3_ grain, reducing its depletion layer. Whereas, other injected electrons recombine with the positive holes inside CuO, extending its depletion layer [1]. Consequently, the depletion layer at the grain-grain boundary is not affected by this reaction (where its depletion thickness increases in p-CuO and decreases in n-In_2_O_3_), and its effect on the gas sensing is negligible. Thus, the sensing property here depends only on CuO and In_2_O_3_ grains. If the ratio of n-type to p-type is 1:1 as in the present case, both effects may cancel each other. Thus, the net change in the resistance will not be noticeable, where the net resistance approximately is the sum of Rp, Rpn, and Rn, as shown in Figure 10b.

**Figure 11:**
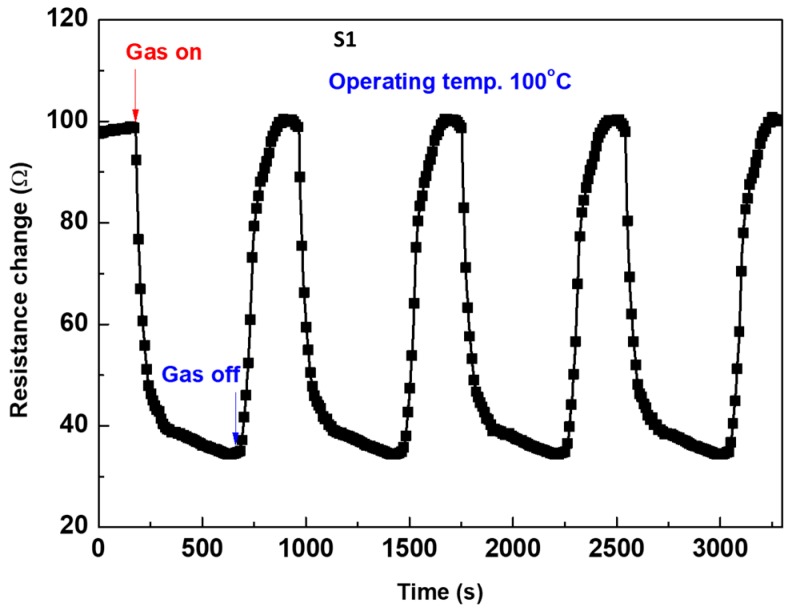
Repeatability sensor signal of the most sensitive S2 layer at 100 °C.

The reversibility of the sensor signal upon exposure to CH_4_ gas is shown in Figure 11. The signal is recorded for the most sensitive sensor fabricated by the S2 sensing layer. The sensor is very sensitive when the gas is switching on/off. The signal is repeated several times, and there is no observed drift. Moreover, the signal is carried out at a low temperature of 100 °C, with quick response and recovery. The time interval for each signal is about 550 s including the steady time for the resistance. This means that the response and recovery times a constant is as short as ~120 s.

## 4. Conclusion

In summary, the CuO-doped In_2_O_3_ nanocomposite was successfully synthesized through the developed vacuum evaporation method. The morphology, structure, and gas sensing features of the prepared films were well characterized. The samples were prepared with different ratios of the weight of Cu and In granules. At low Cu content, the seeds of 1D nanostructure were grown vertically to the substrate surface with needle-like at the tips. However, the excessive Cu content in the composite suppressed the growth of 1D nanostructure. The sensing performance of CuO-mixed In_2_O_3_ film toward methane gas (CH_4_) was examined at low operating temperatures of 50–200 °C. For low Cu content, the fabricated sensors based on CuO-doped In_2_O_3_ showed very good sensing properties at 50 °C. The sensor fabricated by 2.3 mol% CuO-doped In_2_O_3_ exhibited the highest responses at 100 °C, as well as showed good reversibility and stability toward CH_4_ gas. The detection of CH_4_ at low temperatures shows the potential of the present sensors compared to the reported in the literature. However, the preparation of p- and n-type mixed materials by the ratio of 1:1 is not preferred for CH_4_ detection. The gas sensing mechanism was described in terms of the incorporation of Cu in the In_2_O_3_ matrix and the formation of CuO and In_2_O_3_ phases. We can conclude that the present fabricated sensor of low content of Cu has a convenient application for safety control at low temperatures.

## Figures and Tables

**Table 1:** Structural properties of the Cu-mixed In_2_O_3_ nanostructures

XRD Analyses						
Samples	Position obs. (2θ)	h k l	Average Crystallite Size (nm)	Lattice Parameter (Å) (a=b=c)	Unit Cell Volume (Å^3^)	In_2_O_3_ (st)No (04-021-4783) (2θ)
S1	21.3048 30.4071 35.2615 37.4905 60.4823	2 1 1 2 2 2 0 0 4 1 1 4 2 2 6	25.11	10.1514	1046.11	21.3644 30.3924 35.2389 37.4535 60.2597
S2	21.3182 30.4138 35.2888 37.5208 60.5714	2 1 1 2 2 2 0 0 4 1 1 4 2 2 6	25.62	10.1462	1044.51	21.3644 30.3924 35.2389 37.4535 60.2597
S3	21.3287 30.4934 35.4036 37.5412 60.6740	2 1 1 2 2 2 0 0 4 1 1 4 2 2 6	19.17	10.1484	1045.19	21.3644 30.3924 35.2389 37.4535 60.2597

**Table 2:** Recorded frequencies/cm^−1^ in S1, S2, and S3 with their relative intensities: v = very, s = strong, m = medium, w = weak, sh = shoulder.

S1 Raman Shift cm^−1^	S2 Raman Shift cm^−1^	S3 Raman Shift cm^−1^
110 w		110 sh
133 s	133 w	
		161 m
		218 m
		243 w
	251 m	
		282 m
303 vs	303 m	
	337 vs	337 sh
366 s		
	413 w	413 w
495 m		
		562
	593 w	
629 w		622
	635 w	
	680 m	680
